# The University of London and Medical Education

**Published:** 1901-05-18

**Authors:** 


### The University of London and Medical Education.

For many reasons the action to be taken by the newly constituted University of London in regard to medical education is a matter of much interest to the hospitals of the metropolis. It always has been and always will be essential to the medical student that he should have access to the sick for the practical study of disease, and this has, and probably always will render the hospital the dominant factor in the evolution of a medical school. But the medical student of to-day has to learn many things besides what he can pick up in the wards, and the burden of teaching these preliminary and ancillary subjects has to many of the smaller schools become a very serious affair. Hence while everybody admits that the teaching of medicine proper is a matter for the hospitals, and that this work must be distributed among them wherever they exist, a strong feeling has arisen in favour of some form of concentration of the teaching of the earlier subjects, so as to avoid the obvious waste which is involved in every teaching hospital having to possess a fully equipped medical school.

But how far to go, how many subjects to include in the centralising process, and, if any such centralisation should be organised by the University, how to provide for the ordinary non-university student, are questions on which there is much difference of opinion. So far as regards such preliminary scientific subjects as chemistry, physics, and biology, it seems clear that most of the schools would find it a great relief to throw them overboard altogether if only they could do so without the students being snapped up by their opponents. When, however, we come to the intermediate subjects, such as anatomy and physiology, there are many good reasons for believing that it would not be for the advantage of medical education for the teaching of these sciences to be concentrated in one great university school, even if the university had the money wherewith to establish such an institution. Still, though we may admit the benefits of a certain amount of competition and the evils of a complete divorce between the hospitals and the sciences, it is hard to believe that it can be either necessary or advantageous that every hospital which aspires to give technical medical instruction should be forced to provide the very expensive buildings, appliances, and staff which are necessary for the proper teaching of anatomy and physiology.

Many plans have been proposed with the object of concentrating the scientific teaching. Professor Waller suggests, and the suggestion appears a good one so far as it goes, that instead of a sudden concentration of the intermediate studies in one great central university school as has been proposed by some, or in three or four centres, each having the status of a university institution, as is suggested in the scheme of the commissioners, the reduction from twelve (as at present) to a much smaller number of active laboratories should be made to take place gradually and automatically under the constantly acting influence of an effective control by the university, coupled with the establishment as soon as funds may allow of a central university teaching school of the very first class at which a high standard of study and research might be maintained for the imitation of the others.

Practically this scheme is one for the gradual weeding out of the less efficient schools, and however disagreeable they may find the process it would be a good answer to their protests (were the schools alone concerned) to say that it was to the public advantage that the fees now frittered away in the support of a dozen institutions should go to the maintenance of a much smaller number in a much higher state of efficiency. But as it seems to us the hospitals have to be considered as well as the schools, and while any such scheme might usefully tend to concentrate the scientific work, it would tend in a manner anything but useful or desirable to concentrate also the clinical teaching. Notwithstanding the enormous clinical field offered by the London hospitals for the study of disease, it is, we believe, the fact that at some of the medical schools the opportunities for the proper study of the various specialties into which medical work is broken up are even now entirely inadequate, and we cannot hide from ourselves the fact that anything which tends to shut up the smaller schools tends also to withdraw the hospitals connected with them from the clinical field open to the student. Whatever may be the case with the ancillary sciences, the teaching of the technical art of medicine must be personal and practical. The days of great classes are over—one might almost as well read the medical papers—and we should look with great concern upon any movement which should crowd more students into fewer hospitals and so lessen their opportunities for practical work among living patients. This is what would almost of necessity happen if, along with the proposed concentration of the earlier and the intermediate studies in a few great schools, some new arrangements were not made for distributing the students among the various disestablished hospitals. We must always remember, however, that the University is not everything. Until some endowment is forthcoming the whole machinery of medical education is oiled by the fees extracted from ordinary, average students, who mostly go in for the "Conjoint," and until the University caters for "pass" instead of "honours" men the schools will have the whip hand.

